# Histone Phosphorylation in DNA Damage Response

**DOI:** 10.3390/ijms26062405

**Published:** 2025-03-07

**Authors:** Ping Gong, Zhaohui Guo, Shengping Wang, Shufeng Gao, Qinhong Cao

**Affiliations:** 1 Hunan Institute of Microbiology, Changsha 410009, China; azagobio@163.com (Z.G.); victoryprofv@126.com (S.W.); biofighting@163.com (S.G.); 2 College of Biological Sciences, China Agricultural University, No.2 Yuan-Ming-Yuan West Road, Beijing 100193, China

**Keywords:** histone phosphorylation, DNA damage response, γH2AX, kinase, DNA repair

## Abstract

The DNA damage response (DDR) is crucial for maintaining genomic stability and preventing the accumulation of mutations that can lead to various diseases, including cancer. The DDR is a complex cellular regulatory network that involves DNA damage sensing, signal transduction, repair, and cell cycle arrest. Modifications in histone phosphorylation play important roles in these processes, facilitating DNA repair factor recruitment, damage signal transduction, chromatin remodeling, and cell cycle regulation. The precise regulation of histone phosphorylation is critical for the effective repair of DNA damage, genomic integrity maintenance, and the prevention of diseases such as cancer, where DNA repair mechanisms are often compromised. Thus, understanding histone phosphorylation in the DDR provides insights into DDR mechanisms and offers potential therapeutic targets for diseases associated with genomic instability, including cancers.

## 1. Introduction

Eukaryotic cells are frequently subjected to endogenous and exogenous DNA damage, which threatens genome stability and may lead to cellular and systemic imbalances, contributing to the onset of diseases such as cancer [1]. DNA damage arises from a variety of endogenous or exogenous sources, including replication errors, reactive oxygen species, abnormal metabolites, chemical agents, ultraviolet (UV) radiation, and ionizing radiation (IR). To counter these inevitable threats, cells have evolved various DNA damage response (DDR) pathways, which are responsible for detecting, signaling, and repairing DNA damage [2,3]. As the DDR plays a crucial role in maintaining genomic stability, DDR pathway defects can lead to diseases such as premature aging, neurodegenerative disorders, immunodeficiencies, and cancer [1,3,4,5].

The DDR network encompasses a series of intricate signaling and repair mechanisms. The core DDR components include DNA damage recognition, signal transduction, cell cycle regulation, and DNA repair. Initially, cells employ specific sensor proteins to recognize aberrant DNA structures. For example, an excessively long stretch of single-stranded DNA (ssDNA) can accumulate due to DNA unwinding and synthesis uncoupling, where it is rapidly bound by replication protein A (RPA). For instance, an excessively long stretch of single-stranded DNA (ssDNA) accumulates as a result of the uncoupling of DNA unwinding and synthesis. During this process, the DNA is rapidly bound by replication protein A (RPA) [6]. Next, these initial signals and their readers recruit and activate apical kinases such as ataxia telangiectasia-mutated (ATM) and ATM- and Rad3-related (ATR) kinases, which trigger phosphorelay reactions through the mediators to downstream effector kinases, CHK1 and CHK2. These pathways regulate various effector proteins that coordinate DNA repair and induce cell cycle arrest, ensuring sufficient time for repair [2,3].

Eukaryotic cells utilize multiple conserved repair mechanisms depending on the type of DNA damage and cell cycle state. For example, nucleotide excision repair is used to address UV-induced DNA damage [7], whereas mismatch repair (MMR) corrects base-pairing mismatches or insertion/deletion loops formed during DNA replication [8,9,10,11]. Double-strand breaks (DSBs) are among the most severe and lethal forms of DNA damage, often leading to genomic instability. In mammalian cells, DSBs are predominantly repaired via error-prone nonhomologous end-joining (NHEJ) and complementary via error-free homologous recombination (HR) or other pathways in certain circumstances [12,13,14]. NHEJ operates throughout the cell cycle via a template-independent rejoining mechanism with minimal end-processing [15]. In contrast, HR relies on homologous DNA sequences as templates, so it occurs strictly during the S and G2 phases [16,17,18]. In addition, single-strand annealing (SSA) also contributes to DSB repair [13,19]. Furthermore, the DDR is closely linked to apoptosis and senescence pathways. When DNA damage is irreparable, cells undergo programmed cell death to prevent the propagation of the damaged genome. These DDR mechanisms form a complex interconnected network that maintains genomic stability [12,20,21].

Increasing evidence has suggested that post-translational modifications (PTMs) in histones play a pivotal role in the DDR. In eukaryotic cells, chromatin comprises DNA and nucleosomal protein complexes. Each nucleosome core particle consists of an octamer of four core histones (H2A, H2B, H3, and H4), with two molecules of each wrapped in 147 bp of DNA. The linker histone H1 stabilizes chromatin structure by connecting nucleosomes [22]. Histones are essential not only for maintaining DNA structure and genomic stability but also for regulating gene expression. Accordingly, histone PTMs are involved in various biological processes, including gene transcription, DNA replication, chromatin condensation, and DNA damage repair [23,24,25]. Following DNA damage, histones undergo multiple types of PTMs such as phosphorylation, acetylation, methylation, and ubiquitination. These modifications are triggered at damaged sites and facilitate DNA repair through diverse mechanisms [26,27,28].

**Figure 1:**
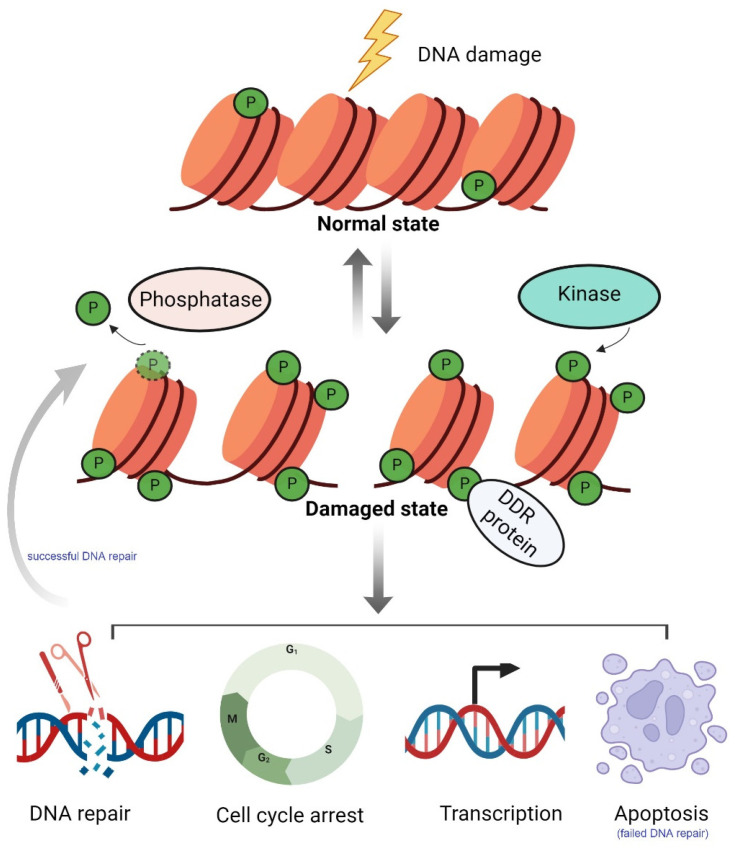
A schematic representation of histone phosphorylation and its roles in the DNA damage response (DDR). Under normal conditions, histones exhibit a low basal level of phosphorylation. When DNA damage occurs, kinases transfer phosphate groups to specific target sites on histones, leading to the accumulation of phosphorylated histones. These phosphorylated histones recruit and coordinate with other proteins involved in the DDR to collectively carry out DDR processes. The main functions of histone phosphorylation in the DDR include facilitating DNA repair, inducing cell cycle arrest, regulating transcription, and promoting apoptosis. Once DNA repair is completed, phosphatases catalyze the removal of phosphate groups, restoring chromatin to its normal state. “P” represents phosphate groups. Created with Biorender.com.

This review primarily highlights the roles of histone phosphorylation in the DDR (Figure 1), as well as its implications in cancer research and therapy. Histone phosphorylation is a key mechanism of epigenetic regulation, dynamically modulating chromatin structure and gene expression by adding phosphate groups (PO₄^3−^) to specific amino acid residues (serine, threonine, and tyrosine) on the N-terminal tails of histones. This dynamic and reversible post-translational modification (PTM) is regulated by specific protein kinases (such as PKA, CDK, and ATR) and counteracted by protein phosphatases (such as PP2A) [29]. As an essential component of chromatin, histone phosphorylation influences chromatin conformation, thereby affecting chromatin accessibility, the binding efficiency of transcription factors, and cellular responses to environmental signals [30,31]. This modification is known to aid in the recruitment of repair factors to damaged sites, the transmission of damage signals, the modulation of chromatin openness and closure, transcription, the regulation of cell cycle progression, and apoptosis, ultimately ensuring effective DNA damage repair and genome stability.

## 2. Histone Phosphorylation in the DNA Damage Response

The phosphorylation sites of different histones play distinct roles in the DDR. This section will provide a detailed analysis of these modifications and their functional significance.

### 2.1. H2A Phosphorylation in the DNA Damage Response

#### 2.1.1. γ H2AX

One of the most well-characterized histone PTMs involved in the DDR is the phosphorylation of the histone variant H2AX. Upon DNA damage, H2AX is phosphorylated at Ser139 by DDR kinases, including ATM and ATR, and DNA-dependent protein kinase (DNA-PK) in mammalian cells. This phosphorylated form is known as γH2AX [32,33,34]. In yeast, where H2AX is absent, corresponding phosphorylation occurs at S129 on H2A (γH2A). ATR is the primary kinase responsible for γH2AX phosphorylation during single-strand damage and replication stress, whereas DNA-PKcs mediate this modification during apoptosis [35,36]. In contrast, DSB-induced H2AX phosphorylation is primarily mediated by ATM or the yTel1 and yMec1 homologs in budding yeast [34,37]. Furthermore, VRK1, a chromatin kinase, is critically involved in the phosphorylation of H2AX at Ser139 in response to DNA damage induced by IR [38].

γH2AX is majorly involved in DDR pathways, including damage signal transduction, NHEJ, and HR [39,40]. This histone modification serves as a biomarker of DNA damage in cancer cells, marking DSBs and facilitating repair protein recruitment, making it one of the most extensively studied histone modifications in the DDR [33]. The importance of γH2AX in DNA repair is highlighted by studies showing that mice lacking H2AX or cells unable to phosphorylate S139 exhibit heightened sensitivity to DNA damage and increased genomic instability [41]. In yeast, the mutation of H2AS129 to a nonphosphorylatable alanine results in hypersensitivity to DNA-damaging agents such as phleomycin and methyl methane–sulphonate (MMS), confirming the critical role of γH2AX in DSB repair. In addition, as γH2AX facilitates sister chromatid recombination, its absence increases reliance on the error-prone SSA repair pathway [39,42].

DSBs are among the most severe forms of DNA damage and can be induced by IR. Upon DSB induction, cells rapidly activate the DDR, recruiting signaling molecules and repair proteins to the damage site to ensure genomic stability and maintain cellular function [43,44]. Initially, γH2AX foci are formed, which provide binding sites for other repair proteins. More specifically, upon DSB occurrence, the Mre11-Rad50-Nbs1 (MRN) complex recognizes the damage and recruits ATM to the site. ATM then phosphorylates H2AX at Ser139, generating γH2AX foci [45]. These foci spread bidirectionally, covering approximately 50 kb in yeast and several Mb in mammals. This extensive γH2AX distribution establishes a signaling platform that facilitates the recruitment and retention of key DDR proteins such as MDC1, p53-binding protein 1 (53BP1), breast cancer 1 (BRCA1), and the MRN complex [39,46,47,48]. MDC1, a critical mediator protein, binds γH2AX via its tandem C-terminal BRCT domains, while it interacts with the FHA domain of P95, a subunit of the MRN complex, and recruits the latter to the DSB site. This interaction amplifies ATM activity, enhancing H2AX phosphorylation and the DDR signal, which facilitates the recruitment of other repair proteins, such as 53BP1 and BRCA1, thus initiating DNA repair [49,50,51].

In addition to IR, other DNA-damaging agents, including UV radiation, chemical mutagens, and replication stress, induce distinct types of DNA lesions, influencing γH2AX activation and propagation and triggering specific DNA damage response (DDR) mechanisms under different genotoxic stress conditions. UV radiation primarily induces pyrimidine dimers, such as cyclobutane pyrimidine dimers (CPDs), which are repaired via nucleotide excision repair (NER). While UV does not directly cause DSBs, in certain contexts, replication fork collapse can lead to secondary DSBs, which in turn trigger γH2AX activation. UV-induced γH2AX is predominantly ATR-dependent and is diffusely distributed in chromatin, contrasting with the focal accumulation observed in IR-induced DSBs [52,53]. Replication stress, arising from factors such as secondary DNA structures or nucleotide depletion agents (e.g., hydroxyurea), primarily activates γH2AX through ATR signaling. Stalled replication forks expose ssDNA, which is recognized by RPA, leading to ATR activation and subsequent H2AX phosphorylation. Unlike the localized γH2AX foci induced by IR, replication stress results in widespread γH2AX distribution, which stabilizes stalled replication forks and prevents their collapse. Additionally, γH2AX facilitates the recruitment of BRCA1 and RAD51, promoting replication fork restart and repair [6,54]. Chemical mutagens, such as alkylating agents (e.g., MMS) and intercalators (e.g., doxorubicin), induce various forms of DNA damage, including base modifications, DNA crosslinks, and strand breaks. The activation of γH2AX varies depending on the lesion type and the corresponding repair pathway. For instance, alkylation damage activates both ATM and ATR, reflecting the integration of DSB formation and replication stress signaling. Meanwhile, DNA crosslinking agents (e.g., cisplatin) promote the γH2AX-mediated recruitment of Fanconi anemia and HR repair factors to facilitate crosslink repair [55,56]. Elucidating the context-dependent dynamics of γH2AX provides critical insights into targeted DNA repair strategies and mechanisms for maintaining genomic stability.

Beyond its role in recruiting DNA repair proteins, γH2AX plays a crucial role in facilitating the recruitment of ATP-dependent chromatin remodeling complexes, such as SWI/SNF and INO80. These complexes utilize ATP hydrolysis to remodel nucleosome architecture, reposition nucleosomes, and mediate histone exchange, thereby dynamically regulating chromatin structure and enhancing DNA repair factor accessibility [57,58]. For instance, upon DNA damage, H2AX phosphorylation serves as a signal for the recruitment of the INO80 complex, which remodels chromatin by repositioning nucleosomes and altering histone composition. Specifically, INO80 facilitates the eviction of canonical H2A and the incorporation of H2A.X, contributing to chromatin reorganization and efficient DNA repair [59]. Similarly, the SWI/SNF complex disrupts nucleosome stability through ATP hydrolysis, promoting histone displacement at specific genomic loci and facilitating access for transcription and repair factors [60]. Through these coordinated mechanisms, γH2AX and chromatin remodeling complexes orchestrate the DDR, ensuring efficient DNA repair and preserving genomic stability.

Although γH2AX is primarily known as a DNA damage repair marker, growing evidence highlights its crucial role in apoptosis, where its phosphorylation contributes to DNA damage sensing and repair and cell death regulation. Unlike the classical DDR, γH2AX formation during apoptosis is not restricted to DNA damage sites but follows distinct spatial and temporal patterns. Initially, it forms a “γH2AX ring” near the nuclear membrane, which expands as apoptosis progresses. This phosphorylation event is closely linked to death receptor activation (e.g., FasL, TRAIL) and H2B dephosphorylation, suggesting a role distinct from DNA repair [61,62]. γH2AX phosphorylation in apoptosis involves DDR kinases such as ATM, DNA-PK, and Chk2. DNA-PK phosphorylates γH2AX early, facilitating apoptosis, while ATM and Chk2 activation induce cell cycle arrest and apoptotic execution [63]. γH2AX also integrates DNA damage and death signals, influencing cell fate by directing repair or apoptosis. Notably, death receptor signaling directly induces γH2AX phosphorylation, reinforcing its role in programmed cell death [64]. In summary, γH2AX extends beyond its conventional role as a DNA damage marker, acting as a key regulator in apoptotic signaling, DNA repair, and cell fate decisions. Its significance as a biomarker in apoptosis research and a potential therapeutic target in cancer treatment warrants further exploration.

Notably, γH2AX can also coordinate the DDR by regulating other types of histone modifications. For instance, γH2AX promotes the recruitment of E3 ubiquitin ligases, including RNF8 and RNF168, to damaged sites, regulating ubiquitylation signaling in DSBs. Upon the occurrence of DNA double-strand breaks, γH2AX is extensively formed at the damage sites, facilitating the recruitment of MDC1. MDC1 subsequently recruits RNF8 to the damaged regions. The mono-ubiquitination of γH2AX, catalyzed by RNF8, provides a binding platform for RNF168, thereby amplifying the ubiquitination signaling cascade and enhancing the recruitment of downstream repair factors [65,66,67]. Moreover, the adaptor protein Rad9, related to 53BP1/Crb2, interacts with γH2AX via its BRCT domain and with methylated H3K79 through its Tudor domain. This specificity enables Rad9 recruitment to the DSB site, where it is phosphorylated by Mec1, triggering a DNA damage checkpoint that delays G1/S progression and allows repair [68,69,70]. In yeast, γH2AX recruits the NuA4 acetyltransferase complex to DSBs. NuA4 mediates H4 hyperacetylation and promotes chromatin relaxation [71,72].

Following DNA repair, γH2AX removal is essential for preventing persistent repair protein recruitment, DNA-damage-induced cell cycle arrest recovery, and chromatin integrity restoration. Two primary mechanisms have been proposed for γH2AX clearance. First, γH2AX can be replaced by unphosphorylated H2A or removed from DSB sites by chromatin remodelers [58,73]. Second, γH2AX is dephosphorylated by various protein phosphatases, regenerating H2AX. In yeast, the HTP-C phosphatase complex regulates H2AS129 dephosphorylation in vivo, enabling DNA damage checkpoint recovery [74]. Similarly, in mammals, phosphatases such as PP2A, Wip1, PP6, and PP4 dephosphorylate γH2AX, allowing effective DNA repair and cell cycle arrest recovery. Among them, PP2A primarily dephosphorylates γH2AX during DSB repair. Comprising a structural subunit A, a regulatory subunit B, and a catalytic subunit C, PP2A directly binds γH2AX at DSB sites, mediating dephosphorylation through its catalytic subunit C. PP2A deficiency results in repair defects and persistent γH2AX accumulation, highlighting the importance of dephosphorylation in postrepair chromatin processing [75,76,77]. Other phosphatases are also involved in γH2AX dephosphorylation. PP6 interacts with the catalytic subunit of DNA-PK to mediate γH2AX dephosphorylation, whereas Wip1 directly induces γH2AX dephosphorylation. PP4 primarily dephosphorylates γH2AX mediated by ATR, enabling DNA damage checkpoint and cell cycle recovery after DNA damage [78,79,80,81]. In summary, the orderly, subsequent γH2AX dephosphorylation is essential for maintaining genomic stability following DNA damage repair.

#### 2.1.2. Other H2A Sites

Although γH2AX is widely used as a DNA damage marker, H2A contains multiple phosphorylation sites that contribute to the DDR apart from serine 139 (S139) phosphorylation.

For instance, tyrosine 142 (Y142) phosphorylation, which is regulated in a DNA-damage-dependent manner, is catalyzed by WSTF kinase. Unlike γH2AX, Y142 phosphorylation is ubiquitously present in cells but decreases significantly upon DNA damage. This inverse relationship is critical for maintaining γH2AX stability at DNA repair foci, as EYA1/3 phosphatase-mediated Y142 dephosphorylation is essential for recruiting repair factors to damaged sites. Failure to dephosphorylate Y142 impairs the accumulation of repair factors and disrupts the DDR [82,83].

In yeast, serine 122 (S122) and serine 129 (S129) on histone H2A are dynamically phosphorylated during DNA damage, contributing to DDR processes. Studies have shown that S122 is critical for cell survival under DNA damage induced by camptothecin, MMS, hydroxyurea (HU), or ultraviolet light. The phosphorylation of S129 in the DDR is dependent on Tel1 and Mec1 kinases, while the phosphorylation of S122 in *Schizosaccharomyces pombe* and *S. cerevisiae* is mediated by Bub1 kinase. Both modifications may facilitate interactions with DDR machinery without altering global chromatin structure. The concurrent phosphorylation of S122 and S129 during DNA damage suggests that they may play synergistic roles in the recruitment or retention of repair factors [29,84,85]. Moreover, a recent study revealed that the phosphorylation of H2A S122, mediated by Bub1 kinase, plays a critical role in regulating chromosome segregation [86].

Recently, DNA-damage-induced H2A phosphorylation at S15, catalyzed by Mec1, was found to be linked to DNA end-resection in yeast. DNA end-resection provides the single-stranded DNA required for HR, thereby potentially assisting in the repair of breaks [87]. Threonine 101 (T101), which is also phosphorylated after DNA damage, is another phosphorylation site. Mutations at this site render cells sensitive to IR, indicating its pivotal role in H2AX-dependent DDR function [88]. Furthermore, the phosphorylation of the threonine 126 (Thr126) residue in H2A.1 is linked to the stability and repair of fragile DNA regions, particularly CAG repeat sequences [89].

These findings collectively highlight the importance of phosphorylation at other H2A sites in DDR regulation. However, the specific regulatory mechanisms of these phosphorylation events in the DDR remain unclear, highlighting the need for further studies to elucidate the mechanisms by which these modifications cooperate with γH2AX to maintain genomic stability following DNA damage.

### 2.2. H3

Histone H3 phosphorylation also plays an important role in the DDR. Studies of key phosphorylation sites such as serine 10 (S10), threonine 11 (T11), and serine 28 (S28), together with their respective kinases, have demonstrated their significance in genomic stability maintenance. These residues are phosphorylated during mitosis to facilitate chromatin compaction [90,91,92,93].

The Aurora kinase family, particularly Aurora B kinase, mediates H3S10 and S28 phosphorylation in the DDR. As a serine/threonine kinase, Aurora B participates in chromosome segregation, cell cycle regulation, and chromatin remodeling. Direct phosphorylation Ser10 in H3 by VRK1 both in vitro and in vivo has been observed [38,94]. G1-phase cells exhibit specific reductions in H3S10 phosphorylation following DNA damage [92]. Concurrent decreases have been demonstrated in additional histone modifications, such as acetylations, accompanied by chromatin condensation. Studies have also suggested the potential crosstalk between H3S10 phosphorylation and other modifications, such as H3K9 acetylation or methylation, collectively affecting chromatin compaction and DNA repair protein recruitment [95,96,97]. These studies suggest dynamic changes in chromatin structure and/or transcriptional repression during the DNA damage response.

Histone H3 threonine 11 (H3T11) phosphorylation also participates in the DDR by regulating chromatin relaxation and DNA repair factor recruitment. Protein kinase C (PKC) or Chk1 kinase typically catalyzes the phosphorylation of H3T11. Following DNA damage, activated PKC phosphorylates H3T11 directly. This modification occurs primarily during the S or G2/M phases, when chromatin structure undergoes significant changes, to facilitate repair protein recruitment Moreover, under environmental stress conditions such as radiation-induced damage, the DDR’s core kinases ATM and ATR may enhance H3T11 phosphorylation indirectly through Chk1 activity modulation, thus influencing chromatin dynamics and DNA repair [91,97]. Moreover, H3T11 phosphorylation mediated by Casein kinase II (CKII) is a key modification for the formation and maintenance of heterochromatin in Neurospora, contributing to genomic stability and the regulation of gene expression [98]. Additionaly, AKT phosphorylates H3-threonine 45 to facilitate the termination of gene transcription in response to DNA damage [99].

Collectively, these H3 phosphorylation events mainly modulate chromatin structure and regulate the recruitment of DNA repair factors, which are essential for effective DNA damage repair. Furthermore, since H3 phosphorylation is crosslinked with other epigenetic modifications, elucidating the mechanisms underlying histone H3 phosphorylation will enhance our understanding of the intricate DDR regulatory networks.

### 2.3. H4

During DNA damage, histone H4 undergoes site-specific phosphorylation by kinases that regulate chromatin structure, repair, and checkpoint regulation.

CKII catalyzes H4 serine 1 phosphorylation (H4S1ph) in yeast in response to UV light-, MMS-, or phleomycin-induced genotoxic stress. This modification contributes to NHEJ [100,101]. H4S1ph accumulates at DSBs, supporting its DNA repair role in humans as well [102]. Interestingly, H4S1ph demonstrates an inverse correlation with H4 acetylation, with its levels decreasing as repair concludes. H4S1ph inhibits the histone acetyltransferase activity of the NuA4 complex in vitro. The association of CKII with the Rpd3S deacetylase complex in vivo suggests that H4S1ph stabilizes newly assembled nucleosomes through acetylation prevention, thereby promoting chromatin restoration [101]. These findings demonstrate the cooperation of histone phosphorylation and deacetylation in mediating NHEJ.

H4Y51, another H4 phosphorylation site, was the first tyrosine phosphorylation modification identified in this histone. This modification, which is catalyzed by the TIE2 kinase, has been linked to NHEJ [103]. Another phosphorylation site, H4T80, also participates in the DDR. H4T80 is phosphorylated by the kinase Cla4 and is recognized by the histone-binding scaffold protein RTT107. The interaction between RTT107 and H4T80p prevents chromatin binding by Rad9, facilitating checkpoint recovery following DNA damage [104].

### 2.4. H2B and H1

In budding yeast, DSBs trigger extensive Tel1 (ATM)- and Mec1 (ATR)-mediated H2A phosphorylation near break sites, leading to γ-H2AX formation. Similarly, DNA damage triggers Tel1- and Mec1-mediated H2B phosphorylation at T129. The distribution of H2BT129p mirrors that of γ-H2AX in yeast, forming large domains around break sites. Notably, the absence of γ-H2AX impaired γ-H2B formation [105] suggests a potential cooperation between these modifications in the DDR. In mammalian cells, DSBs induce H2B phosphorylation at serine 14 by MST1 kinase [106]. In addition, H2BS14p is a hallmark histone modification closely associated with chromatin remodeling and apoptosis [107]. However, the regulatory mechanisms and functions of this modification remain incompletely understood. Current understanding of DSB-induced H2B phosphorylation remains limited, particularly regarding specific enzymes and recognition mechanisms.

The phosphorylation of the linker histone H1 has also been found to be associated with the DNA damage response. Studies show that a H1 subtype, H1.2, is phosphorylated at threonine 145 (H1.2T145p) in the p53-dependent DDR. Under normal conditions, unphosphorylated H1.2 interacts with p53 to keep its target genes repressed. Following DNA damage, DNA-PK phosphorylates H1.2 at T145, disrupting its interaction with p53. This promotes the recruitment of chromatin-remodeling complexes and transcription factors to p53 target promoters, ultimately activating the p53 transcriptional program to maintain genome stability [108,109].

In summary, apart from γH2AX, the phosphorylation of other sites also plays crucial roles in the DDR, including facilitating DNA damage repair, regulating the cell cycle, modulating chromatin dynamics, and promoting apoptosis. Unraveling these mechanisms will enhance our understanding of complex DDR regulatory networks and offer new avenues for the diagnosis and treatment of related diseases.

## 3. Histone Phosphorylation in Cancer Research and Therapy

Histone phosphorylation is crucial for the DDR and genome stability maintenance, holding significant therapeutic implications. Research on histone phosphorylation in human cancers has not only uncovered its roles beyond DDR pathways but has also facilitated its application in cancer therapy, leading to ongoing clinical trials and the development of approved drugs targeting histone phosphorylation.

### 3.1. Histone Phosphorylation in Cancer Research

Studies have demonstrated a strong correlation between abnormal histone phosphorylation and cancer development. For example, colorectal cancer tissues exhibit elevated mRNA levels of H2AX and increased γH2AX expression compared to those in normal tissues, correlating with aggressive tumor behavior and poor patient survival [110,111,112]. Notably, H2AX phosphorylation levels increase significantly during DNA fragmentation and apoptosis [39]. The relationship between H2AX expression and microsatellite instability, a carcinogenic mechanism driven by mismatch repair defects, further emphasizes the connection between γH2AX and cancer progression. In colorectal cancer, reduced H3 Ser10 (H3S10) and Y74 and Y272 phosphorylation levels mediated by T-LAK cell-originated protein kinases (TOPKs) promote tumor development [113]. Aurora B, which is critical for H3 phosphorylation and chromosome segregation, is overexpressed in various cancers, including colorectal and breast cancers [114]. In prostate cancer cells, androgen stimulation activates kinases PKCβ and PRK1, which phosphorylate H3Thr6 and H3Thr11, respectively [115,116]. In addition, Mst1 kinase phosphorylates H2AX, and its overexpression induces apoptosis in HELA cells via H2AXSer139p [117].

Histone phosphorylation is intricately linked to transcriptional regulation, particularly that of genes involved in cell cycle control and proliferation [118]. For instance, Janus kinase 2 (JAK2) phosphorylates H3Tyr41, disrupting the interaction between heterochromatin protein 1α (HP1α) and chromatin. This loss of HP1α binding leads to the constitutive activation of the JAK2 signaling pathway, including the proto-oncogene imo2, thereby driving oncogenesis. JAK2-mediated H3Y41 phosphorylation facilitates the transcriptional activation of diverse gene sets in a cancer-patient-specific manner [119,120]. Furthermore, the phosphorylation of H3 at Ser10 and Ser28 and H2B at Ser32 is associated with epidermal growth factor (EGF)-mediated gene transcription. UVB radiation exposure increases H3Ser10p and H2BSer32p levels, upregulating the expression of proto-oncogenes such as c-myc, c-fos, and c-jun, whereas H3Ser28p specifically regulates c-fos and α-globin activation [121,122,123]. The levels of H2BSer32 phosphorylation, which is mediated by RSK2, are significantly elevated in skin cancer cells [124].

### 3.2. Histone Phosphorylation in Cancer Therapy

#### 3.2.1. γ H2AX in Cancer Therapy

In addition to its role as a DNA damage marker, γH2AX has gained importance in cancer research and treatment. It has been widely used to evaluate radiotherapy and chemotherapy efficacy, predict tumor cell sensitivity to treatment, and serve as a potential therapeutic target [39,111,125,126]. Cancer cells, characterized by genomic instability, exhibit alterations in γH2AX levels that closely correlate with therapy response. Some cancer cells evade treatment by enhancing their DNA repair capabilities, with increased γH2AX levels facilitating the effective repair of chemotherapy- or radiotherapy-induced DNA damage, thus contributing to treatment resistance [127,128].

γH2AX demonstrates utility in auxiliary diagnosis and prognosis monitoring across multiple diseases. The high-throughput mass spectrometry quantification of γH2AX changes has been used to detect DNA damage in human peripheral blood cells exposed to low-dose environmental IR [129]. In addition, γH2AX levels in circulating tumor cells in chemotherapy patients serve as prognostic markers [130]. In reproductive cell research, γH2AX has been used for assessing DNA damage and repair capacity in sperm and oocytes and is involved in maintaining embryonic stem cell self-renewal [131,132]. Glycolytic metabolite pyruvate has been shown to promote FACT-complex-mediated γH2AX loading onto chromatin, enhancing DNA damage signaling and repair, thereby supporting glioblastoma cell survival after DNA damage. These findings provide new strategies for improving the efficacy of glioblastoma multiforme treatment [133]. H2AX has emerged as a crucial target for therapeutic strategy development, with drugs that modulate its function undergoing investigation from preclinical studies to clinical trials. Synthetic lethality approaches, particularly in combination with chemotherapy or radiotherapy, have been explored by targeting key enzymes in DDR pathways, such as ATM, ATR, and DNA-PK. These strategies aim to exploit vulnerabilities in cancer cells with defective DNA repair mechanisms, thereby enhancing treatment specificity and efficacy [134,135].

#### 3.2.2. Clinical Trials and Approved Drugs Targeting Histone Phosphorylation

Histone phosphorylation plays a central role in the DDR, making it a promising therapeutic target in cancer treatment. Several drugs targeting histone kinases or related pathways are currently under clinical investigation, with some already receiving regulatory approval for cancer therapy.

JAK2 inhibitors, which modulate histone H3 tyrosine 41 (Tyr41) phosphorylation and chromatin accessibility, have gained significant attention in hematologic malignancies. Ruxolitinib, a JAK1/2 inhibitor, reduces histone phosphorylation by blocking the JAK-STAT pathway, thereby suppressing inflammation-associated gene expression. This agent has been approved for the treatment of myelofibrosis, hemophagocytic lymphohistiocytosis, and polycythemia vera [136,137,138]. Aurora kinases, which regulate mitosis through histone H3 phosphorylation, have emerged as key therapeutic targets. Alisertib, an Aurora A kinase inhibitor, has shown promising efficacy in clinical trials for solid tumors and hematologic malignancies, including a phase II trial in patients with castration-resistant and neuroendocrine prostate cancer [139,140,141]. Meanwhile, the Aurora B kinase inhibitor Barasertib is undergoing active evaluation for the treatment of leukemia and other malignancies [142,143]. Additionally, VRK1 kinase phosphorylates histone H3 at Ser10 and plays a crucial role in chromatin remodeling, making it a potential drug target. Preclinical studies suggest that VRK1 inhibition can sensitize cancer cells to DNA-damaging agents, and ongoing research is exploring its therapeutic potential [144].

Histone phosphorylation also interacts with other epigenetic modifications, supporting the development of combination therapy strategies. For example, BET inhibitors and HDAC inhibitors (e.g., Vorinostat, Panobinostat) are being investigated in combination with kinase inhibitors to enhance anticancer efficacy [145]. Notably, the combination of Alisertib and Fulvestrant has demonstrated encouraging clinical activity in breast cancer patients [146].

Despite significant advancements in targeting histone phosphorylation, challenges remain, particularly regarding the specificity of kinase inhibitors and potential off-target effects. The integration of histone phosphorylation research with therapeutic development is driving innovations in oncology. Future research will focus on combining histone phosphorylation inhibitors with immunotherapy and precision medicine to further enhance their therapeutic potential in cancer treatment.

## 4. Conclusions and Perspectives

This review summarizes the research progress on histone phosphorylation in the DDR (Table 1) and its application significance in cancer research and therapy.

Histone phosphorylation plays a crucial role in the DDR by facilitating chromatin remodeling, recruiting damage-repair proteins, mediating signal transduction, and regulating cell cycle checkpoints. Notably, H2AX phosphorylation at Ser139 (γH2AX) represents a hallmark of the DDR, following DNA DSBs. γH2AX serves as an early marker of the DDR and plays a pivotal role in detecting and repairing DNA damage. γH2AX formation and dephosphorylation are the most extensively studied histone phosphorylation events [39,41,128]. γH2AX participates in repair pathways such as NHEJ and HR and provides a critical tool for cancer diagnosis, treatment evaluation, and prognosis monitoring. In addition, histone phosphorylation interacts with other epigenetic modifications, such as methylation and acetylation, to coordinate DDR regulation.

Despite significant progress in elucidating the relationship between histone phosphorylation and the DDR, many critical questions remain unanswered. For instance, first, the precise molecular mechanisms underlying histone phosphorylation in the DDR require clarification, particularly regarding the specific roles of different phosphorylated histones in DNA damage recognition, signaling, and repair. Second, the mechanisms by which histone phosphorylation interacts with other epigenetic modifications to orchestrate the DDR require further investigation. Third, the reversible histone phosphorylation/dephosphorylation cycle is intimately embedded throughout the full round of the DDR process, i.e., activation/deactivation (recovery). Therefore, a challenging task is to resolve histone phosphorylation and its related PTM levels in a precise, quantitative, spatio-temporal manner. Furthermore, the association between aberrant histone phosphorylation and cancer initiation and progression, as well as its potential as a therapeutic target, requires extensive clinical and fundamental research.

In eukaryotic organisms, histone variants and chromatin architecture play a pivotal role in modulating the DDR. The phosphorylation patterns of histones exhibit adaptive divergence, particularly between radiation-sensitive and radiation-resistant species, reflecting their distinct DNA repair mechanisms. In radiation-sensitive organisms, such as insects, H2AX phosphorylation occurs rapidly upon DSB, facilitating the activating the ATM-dependent signaling cascade, which orchestrates cell cycle checkpoint activation and DNA repair [147,148]. This “speed-prioritized” immediate response relies on evolutionarily conserved signaling pathways and may be further reinforced by selective environmental pressures. In contrast, radiation-resistant species, such as rotifers, exhibit more tightly regulated histone phosphorylation, with reductions in modifications such as H3T11ph potentially contributing to chromatin compaction and damage signal attenuation. This regulation may restrict chromatin decompaction, thereby limiting the propagation of DNA damage signals. Consequently, these organisms tend to favor alternative repair pathways, such as HR or NER, potentially minimizing mutagenic risks and adopting an “accuracy-prioritized” repair strategy [149,150]. The evolutionary divergence of histone phosphorylation patterns underscores adaptive responses to environmental stressors. Elucidating the regulatory mechanisms underlying these modifications not only enhances our understanding of the evolutionary dynamics of DNA repair but also provides valuable insights for biomedical and biotechnological applications.

Future research directions should focus on several key areas: employing advanced proteomics and genomics technologies to systematically identify and elucidate the roles and regulatory networks of histone phosphorylation in the DDR, developing cell and animal models to investigate the mechanism by which aberrant histone phosphorylation affects genomic stability and tumorigenesis, developing drugs targeting histone phosphorylation modifications, and evaluating their potential efficacy and safety in cancer therapy. These efforts are expected to uncover the intricate roles of histone phosphorylation in the DDR and cancer, paving the way for novel diagnostic, preventive, and therapeutic strategies for tumor management.

## Figures and Tables

**Table 1:** A summary of histone phosphorylation in the DDR discussed in this review.

Histone Phosphorylation Sites	Kinases	Function	Refs.
H1.2-T145	DNA-PK	chromatin remodeling; p53 transcription	[108,109]
H2A.1-T126	unknown	affecting the stability and repair of fragile DNA regions	[89]
H2A-S122	Bub1	DNA repair; chromosome segregation	[84,86]
H2A-S15	Mec1	influencing chromatin dynamics and DNA end-resection	[87]
H2AX-S139 (H2A-S129 in yeast)	ATM, ATR, DNA-PK	DNA repair; damage-signal transduction; transcription; checkpoint regulation; apoptosis	[39,43,44,51,57,70,117]
H2AX-T101	unknown	reducing cells’ sensitivity to IR	[88]
H2AX-Y142	WSTF	DNA repair	[82,83]
H2B-S14	MST1	chromatin remodeling and apoptosis	[106,107]
H2B-T129	Mec1/Tel1	unclear, possibly coordinated with function of γH2AX	[105]
H3-S10	Aurora-B	transcription; modulating chromatin structure	[92,93,97,121]
H3-S28	MSK1	modulating chromatin structure; transcription	[93,123]
H3-T11	CHK1, CKII	DNA repair; transcription; maintenance of heterochromatin	[91,97,98]
H3-T45	AKT	transcription	[99]
H4-S1	CKII	DNA repair	[100,101]
H4-T80	Cla4	checkpoint regulation	[104]
H4-Y51	TIE2	DNA repair	[103]

## Abbreviations

DDR	DNA damage response
53BP1	p53-binding protein 1
ATM	ataxia telangiectasia-mutated
ATR	ATM- and Rad3-related
BRCA1	breast cancer 1
CKII	casein kinase II
DNA-PK	DNA-dependent protein kinase
DSB	double-strand break
EGF	epidermal growth factor
HR	homologous recombination
NER	nucleotide excision repair
HU	hydroxyurea
IR	ionizing radiation
JAK2	Janus kinase 2
CPDs	cyclobutane pyrimidine dimers
MMR	mismatch repair
MMS	methyl methane–sulphonate
MRN	Mre11-Rad50-Nbs1
NHEJ	nonhomologous end-joining
PTM	post-translational modification
RPA	replication protein A
SSA	single-strand annealing
ssDNA	single-stranded DNA
TOPK	T-LAK cell-originated protein kinase
UV	ultraviolet
DDR	DNA damage response
53BP1	p53-binding protein 1
